# *Candida albicans* Strains Adapted to Caspofungin Due to Aneuploidy Become Highly Tolerant under Continued Drug Pressure

**DOI:** 10.3390/microorganisms11010023

**Published:** 2022-12-21

**Authors:** Farha Husain, Anshuman Yadav, Sudisht K. Sah, Jeffrey J. Hayes, Elena Rustchenko

**Affiliations:** Department of Biochemistry and Biophysics, University of Rochester Medical Center, Rochester, NY 14642, USA

**Keywords:** *Candida albicans*, caspofungin, echinocandins, cross-adaptation, evolution of drug tolerance, aneuploidy

## Abstract

*Candida albicans* is a prevalent fungal pathogen of humans. Understanding the development of decreased susceptibility to ECN drugs of this microbe is of substantial interest, as it is viewed as an intermediate step allowing the formation of *FKS1* resistance mutations. We used six previously characterized mutants that decreased caspofungin susceptibility either by acquiring aneuploidy of chromosome 5 (Ch5) or by aneuploidy-independent mechanisms. When we exposed these caspofungin-adapted mutants to caspofungin again, we obtained 60 evolved mutants with further decreases in caspofungin susceptibility, as determined with CLSI method. We show that the initial adaptation to caspofungin is coupled with the adaptation to other ECNs, such as micafungin and anidulafungin, in mutants with no ploidy change, but not in aneuploid mutants, which become more susceptible to micafungin and anidulafungin. Furthermore, we find that the initial mechanism of caspofungin adaptation determines the pattern of further adaptation as parentals with no ploidy change further adapt to all ECNs by relatively small decreases in susceptibility, whereas aneuploid parentals adapt to all ECNs, primarily by large decrease in susceptibilities. Our data suggest that either distinct or common mechanisms can govern adaptation to different ECNs.

## 1. Introduction

The human fungal pathogen *Candida albicans* is the most common cause of systemic candidiasis. Along with infections by non-*albicans* species, *C. albicans* increasingly displays resistance to antifungals [1,2,3,4,5]. *C. albicans* resistance to drugs from the front-line class of echinocandins (ECNs) is exclusively due to point mutations in the *FKS1* (orf19.2929) gene, which encodes for a catalytic subunit of 1,3-β-glucan synthase [6]. These mutations are clustered in two regions, HS1 and HS2, encompassing residues 641 to 649, and 1345 to 1365, respectively [7], and cause dramatic elevation of *C*. *albicans* minimum inhibitory concentration (MIC) values to ECNs [6]. However, many clinical isolates display a wide range of ECN drug susceptibilities with MIC increases up to the clinical breakpoint in the absence of *FKS1* resistance mutations [8,9,10,11], a physiological state that we hereafter refer to as adaptation or tolerance. As drug tolerance is thought to be a step leading to development of clinical resistance due to *FKS1* mutations, understanding mechanisms of tolerance is of considerable importance [12].

Previously, several laboratories reported that caspofungin-adapted mutants are easily generated with *C. glabrata* or *C. albicans* in vitro on agar plates that are supplemented with caspofungin (reviewed in [12]). Such mutants have no mutations in the clinically important *FKS1* gene and typically exhibit 2- to 8-fold MIC increases, *C. albicans* [13], or up to 32-fold MIC increase, *C. glabrata* [14]. It was also demonstrated with laboratory-generated *C. albicans* mutants that aneuploidy of either chromosome 5 (Ch5) or Ch2, as well as yet undefined aneuploidy-independent mechanism(s) are responsible for increased caspofungin MICs (reviewed in [12]). We consider these caspofungin-tolerant mutants as models of *C. albicans* clinical isolates with increased MICs. The purpose of this paper is to assess the ability of aneuploidy-dependent and independent mechanisms to support the further evolution of tolerance in the presence of caspofungin.

## 2. Materials and Methods

### 2.1. Strains, Media and Growth Conditions

*C. albicans* caspofungin-adapted mutants used in this study are listed in Table S1 (also see Table 1). Chromosomal condition of these mutants was examined by pulse-field gel electrophoresis. DNA-seq confirmed the chromosomal condition of SMC60-2-5 and JMC160-2-5 [13].

Cells were stored in 25% *vol*/*vol* glycerol solution at −80 °C to interrupt cellular metabolism and, thus, prevent induction of genetic instability [15].

YPD medium was prepared with 1% yeast extract, 2% peptone, 2% dextrose. To prepare solid medium, 2% (*w*/*v*) agar was added. Caspofungin (Merck Sharp and Dohme Corp., Kenilworth, NJ, USA); anidulafungin (Pfizer Inc., New York, NY, USA); or micafungin (MedChem Express LLC, Monmouth Junction, NJ, USA) were handled according to manufacture recommendations and added when needed.

Primers used in this study are listed in Figure S1.

### 2.2. Broth Microdilution Assay to Determine Minimum Inhibitory Concentration (MIC)

Broth microdilution assay was performed according to Clinical and Laboratory Standards Institute (CLSI) reference M27-A3 broth microdilution method for yeasts [16] with some modifications. Briefly, assay was conducted with YPD medium in a total volume of 200 µL. A series of two-fold dilutions of caspofungin, anidulafungin or micafungin were prepared directly in 96-well, flat-bottom, polystyrene, microtiter plates. 10^3^ cells in 100 µL of YPD medium were inoculated in each well to have a total volume of 200 µL. Each strain was tested in duplicate or triplicate on a microtiter plate. Parental strain was included on each plate. Plates were incubated at 35 °C and monitored for 48 h. Control well without cells was used to subtract background and no drug control well was used for normalization. The optical densities were measured with Spark Multimode Microplate Reader (TECAN, Zurich, Switzerland) at 600 nm. Normalized readings were generated in Microsoft Excel 2016 and presented as heat maps. Each assay was repeated three times in independent experiments.

### 2.3. In Vitro Evolution of Strains

Clump of *C. albicans* cells was removed from −80 °C frozen stock, streaked on YPD plates and incubated at 37 °C until colonies containing ~10^5^ cells per colony. Colonies were collected and resuspended in 1 mL of saline water (0.9% NaCl), as recommended in [17]. An appropriate adjustment of cell density in suspension was performed with the aid of haemocytometer. After that, we followed the experimental design by Balashov et al. [18]. Namely, ~10^8^ colony-forming units (CFU) were spread on each of three YPD plates supplemented with caspofungin at twice concentration of MIC for each strain, as well as on three drug-free control YPD plates. In addition, ~10^3^ CFU were spread on each of three drug-free control YPD plates. Plates were incubated at 37 °C for 2 to 3 days. Each colony that evolved on drug plates was purified by subcloning on a fresh YPD plate containing an amount of drug equivalent to that used for initial selection. Glycerol stocks of evolved colonies were prepared and stored at −80 °C. Further, strains from frozen glycerol stocks were evaluated with broth microdilution assays for their MICs.

Frequencies of colonies with increased tolerance to caspofungin were calculated by dividing the number of colonies with increased MIC on each of three drug plates by the starting inoculum plated, ~10^8^ CFU, and subsequently averaging three values.

### 2.4. Sequencing of FKS1 Hot Spots HS1 and HS2 Regions

Cells were removed from −80 °C frozen stock, streaked on YPD plates and incubated at 37 °C until colonies contained ~10^5^ cells. Colonies were collected and genomic DNA was extracted using Cold Spring Harbor Protocol [19]. Q5 high-fidelity DNA polymerase (New England BioLabs, Ipswich, MA, USA) was used to amplify the *FKS1* HS1 and HS2 regions with the following PCR conditions: an initial denaturation at 98 °C for 30 s followed by 34 cycles of 98 °C for 10 s, 56 °C for 20 s, and 72 °C for 30 s and then a final 5 min final extension at 72 °C. Amplicons were sequenced by Azenta Genewiz (Plainfield, NJ, USA) (see the bottom of Figure S1 for the cartoon of regions and position of primers). NCBI basic local alignment search tool was used for analysis of sequencing results.

## 3. Results and Discussion

### 3.1. Caspofungin-Adapted Parental Mutants Have Decreased Susceptibility to Caspofungin Only

**Figure 1:**
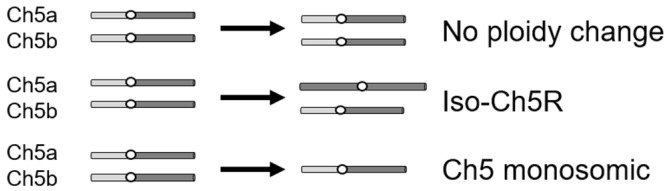
Cartoon presenting three types of caspofungin-adapted mutants that were used in this work to generate caspofungin-evolved mutants. Shown is condition of only Ch5 in each mutant type, as explained on the right.

To study the *C. albicans* evolutionary path in response to caspofungin, we used drug-adapted mutants we previously derived from strains JRCT1 or SC5314 by direct selection on solid medium supplemented with lethal amounts of caspofungin (Table S1; [13]). Tolerance in these mutants is associated with remodeling of the cell wall, characterized by increased chitin and decreased β-glucan, as well as with expression changes of three *FKS* genes in the absence of canonical resistance mutations in the clinically relevant gene *FKS1*. Three different mechanisms are responsible for the caspofungin tolerance in the above mutants: (i) aneuploidy-independent mechanisms where mutants remain normal diploids; (ii) combination of one normal Ch5 and one iso-Ch5R having two right arms, and (iii) loss of one copy of Ch5, resulting in Ch5 monosomy (Figure 1) [13]. Here, we used two representative caspofungin-adapted mutants representing each of the three mechanisms, i.e., no ploidy change, JMC200-2-5 and JMC160-2-5; iso-Ch5R, JMC120-1-6 and JMC120-2-5; and Ch5 monosomy, SMC60-3-4 and SMC60-2-5 that arose from two different genetic backgrounds (see Table S1). We confirmed increased caspofungin MICs (Materials and Methods) of the six caspofungin-adapted mutants vs. their parental strains, which were consistent with that first reported [13] (Figure S2, Table S1).

We next asked whether the initial caspofungin adaptation is coupled with adaptation to two other ECNs, micafungin and anidulafungin. As presented in Figure S2, Table S2 and summarized in Table 1, different classes of caspofungin-adapted mutants (Figure 1) show different susceptibility levels to the other ECNs. Specifically, euploid mutants, referred to here as “no ploidy change” became adapted to all three ECNs. In contrast, Ch5 aneuploid mutants exhibited increased susceptibility to micafungin and anidulafungin. Cross-adaptation of the no ploidy change mutants to ECNs is reminiscent of recently reported *C. albicans* mutants adapted to the new ECN rezafungin [20], which also became more tolerant to caspofungin, micafungin, and anidulafungin [21]. However, the increased susceptibility phenotypes of mutants with Ch5 aneuploidy are reminiscent of previously reported CRS-MIS (Caspofungin Reduced Susceptibility—Micafungin Increased Susceptibility) phenotypes of caspofungin-adapted *C. glabrata*, due to altered membrane sphingolipids [14,22]. These data raised the possibility that distinct mechanisms can lead to adaptation to specific ECN drugs or groups of drugs in *C. albicans*.

### 3.2. Generation and Properties of Strains That Evolved from Caspofungin-Adapted Mutants

**Figure 2:**
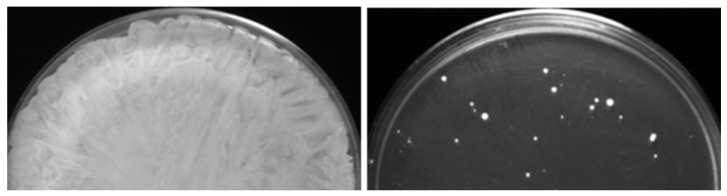
Generation of caspofungin-evolved mutants from caspofungin-adapted parental mutants. (**Left**) Confluent growth was obtained after plating ~10^8^ CFUs of the caspofungin-adapted parent on control YPD medium. (**Right**) Only some colonies, i.e., caspofungin-evolved mutants, were obtained after plating ~10^8^ CFUs of the caspofungin-adapted parent on YPD medium supplemented with twice the MIC of caspofungin concentration.

**Figure 3:**
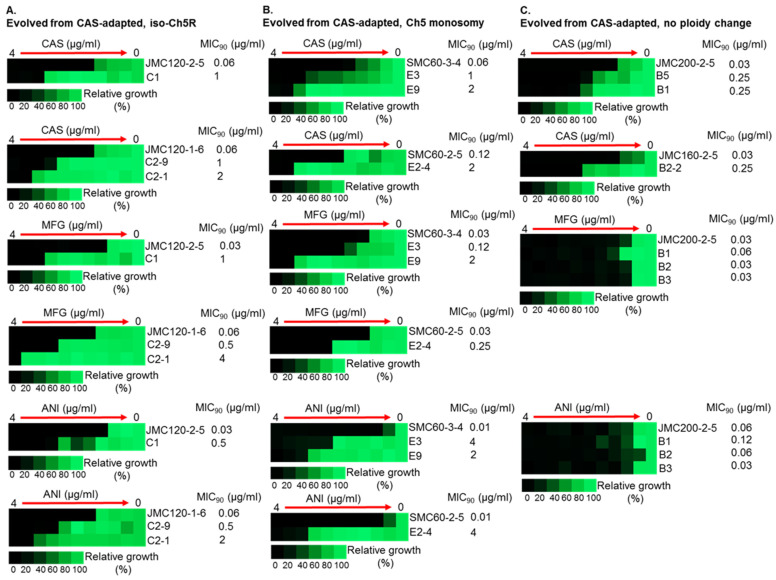
Increased MICs of caspofungin-evolved mutants and cross-adaptation of mutants with alterations of Ch5 to other ECNs. The heat maps represent growths of various *C. albicans* strains by broth microdilution assay. CAS, MFG and ANI refer to caspofungin, micafungin and anidulafungin, respectively. Shown are representative mutants that evolved from CAS-adapted JMC120-1-6 and JMC120-2-5 having iso-Ch5R, (**A**); SMC60-3-4 and SMC60-2-5 having Ch5 monosomy, (**B**); and JMC200-2-5 and JMC160-2-5 with no ploidy change, (**C**). Assay was performed according to CLSI method in YPD medium including maximum CAS, MFG and ANI concentration of 4 μg/mL and two-fold serial dilutions. 10^3^ cells were inoculated in each well in either duplicates or triplicates (technical replicates). Plates were incubated at 35 °C for 24 h. Control wells without drug or without cells were included. No cells control was used to subtract background. No drug control was used for normalization. Color bar for % growth is presented for each panel underneath. MICs were measured as 90% growth inhibition relative to a drug free control. Note that mutants evolved from no ploidy change parentals, often show no cross adaptation with other ECNs.

The six caspofungin-adapted mutants were plated on solid medium supplemented with twice the caspofungin MIC concentration for each strain. All 106 colonies that grew on the drug plates (see example in Figure 2) were collected and checked for their caspofungin MICs with the broth microdilution method. Sixty (of the 106) colonies acquired increased caspofungin MICs, with values ranging from 2- to 256-fold compared to the MICs of the parental caspofungin-adapted mutants (Table 2 and Table S2; Figure 3 and Figure S3). We designated the 60 corresponding strains as evolved mutants. Frequencies of the evolved mutants ranged from 8.6 × 10^−8^ to 4.5 × 10^−7^.

Most important, evolved mutants with higher (≥16-fold) increases in caspofungin MIC levels also displayed high tolerance to micafungin and anidulafungin, exhibiting increases in MICs ranging from 16- to 256-fold for these ECNs (Table 2 and Table S2, Figure 3).

We noted that many adapted mutants exhibited very high MIC values, covering a range of 1–4 µg/mL for all three ECNs tested, with 23, 17, and 16 mutants exhibiting MIC values in this range for caspofungin, micafungin, and anidulafungin, respectively. These values are at or above the currently established breakpoint (BP) values for resistance, ≥1 µg/mL by CLSI, for *C. albicans* and caspofungin/micafungin/anidulafungin [23]. Our limited analysis of possible mechanisms behind the larger increases of MICs included Sanger sequencing of HS1 and HS2 regions of the clinically important gene *FKS1*. The sequencing was performed with four representative evolved mutants (C1, C2-1, E9, and E2-4) that were derived from caspofungin-adapted parentals with aneuploid Ch5 and acquired high MIC increases for caspofungin (8-, 32-, 64- and 32-fold respectively), micafungin (32-, 64-, 64- and 8-fold respectively) and anidulafungin (16-, 32-, 128- and 256-fold respectively) (Table S2 and Figure S3). Importantly, in all of these cases we found no *FKS1* resistance mutations. Control sequencing of B1 (exhibited 4-, 2-, and 2-fold MIC increase for caspofungin, micafungin and anidulafungin respectively) and B2-20 (8-fold MIC increase for caspofungin, while no change in MIC observed for micafungin and anidulafungin), as expected, revealed no *FKS1* mutations. These results demonstrate that *C. albicans* has the ability to coordinately and highly increase tolerance to multiple ECNs independently of Fks1p. Further studies with animal models are required to assess the impact of the high MICs of such mutants upon their level of tolerance/resistance in vivo.

**Figure 4:**
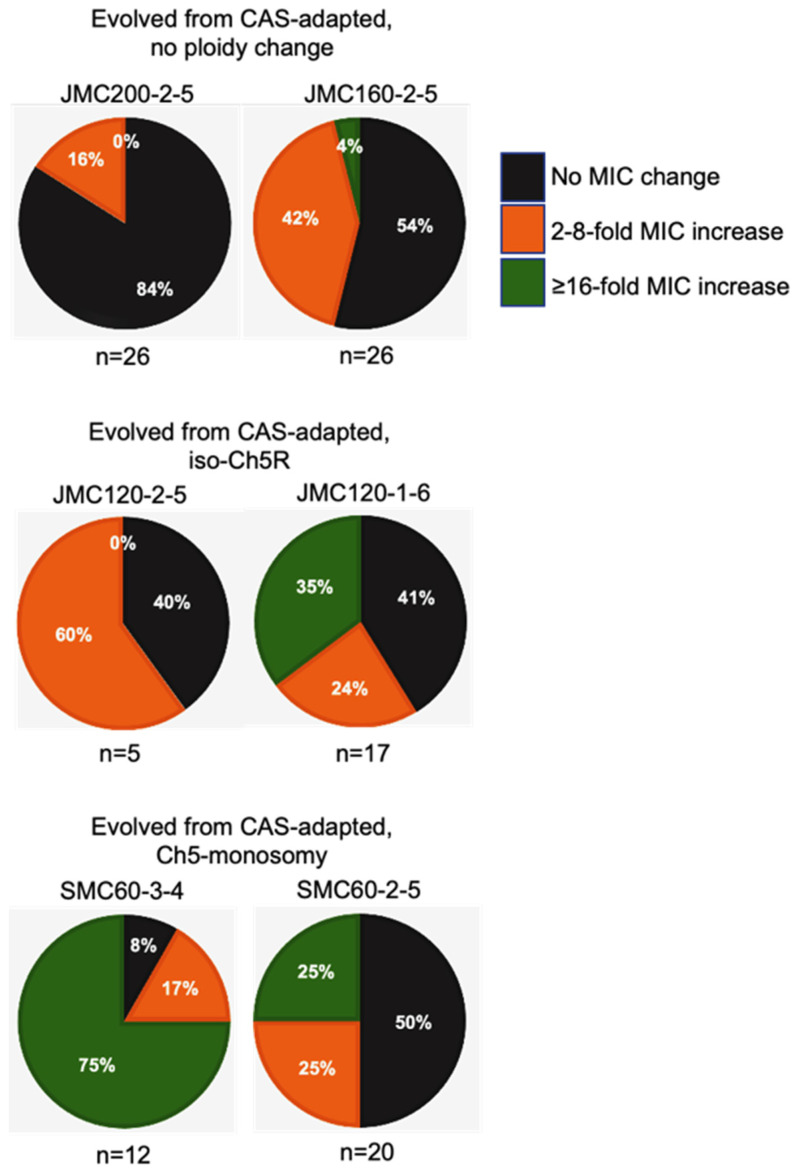
Pie charts depicting the percentage of caspofungin-evolved mutants from each class of caspofungin-adapted mutants, no ploidy change, JMC200-2-5 and JMC160-2-5; iso-Ch5R, JMC120-1-6 and JMC120-2-5; and Ch5 monosomy, SMC60-3-4 and SMC60-2-5. Evolved mutants are presented according to their caspofungin MICs. Black, orange and green colors are representing the proportion of colonies with no change in MIC, 2–8-fold increase, and ≥16 fold increase in MIC, respectively, as compared to their caspofungin-adapted parental strains.

Intriguingly, the levels of high caspofungin MIC increases distributed non-randomly among evolved mutants based on parental origin. Parental strains with no ploidy change produced evolved mutants with predominantly relatively moderate 2- to 4-fold increases in MICs, with values under the BP for resistance. Some of these mutants also showed relatively moderate increase of micafungin and anidulafungin MICs. In contrast, practically all mutants that exhibited high increases of caspofungin MICs ranging from ≥16-fold were evolved from parentals containing different Ch5 aneuploidies, as summarized in Table 2 and Figure 4. Interestingly, the high increases of caspofunign MICs for evolved mutants derived from aneuploid parentals were all associated with cross-adaptation, as these strains also exhibited up to 128 to 256-fold increases in MICs to micafungin and anidulafungin.

As there were a number of colonies grown on drug plates that did not exhibit decreased caspofungin susceptibility when tested with the broth microdilution method, we tested two representative colonies B4 and B2-3 with spot assays on caspofungin, micafungin and anidulafungin drug plates. B4 showed no difference in the growth vs. parental strain, whereas B2-3 grew substantially better than the parental strain, hence acquired tolerance to ECNs (data not shown). We conclude that at least some of 45 mutants that did not exhibit adaptation to caspofungin by broth microdilution did, in fact, acquire decreased ECN susceptibilities.

We speculate that one of the putative mechanisms responsible for MIC increases could be transcriptional changes of multiple genes that have a role in ECN susceptibility (see [24]). It will be interesting in future experiments to compare composition of cell walls in evolved mutants vs. their parents, as well as to analyze genome-wide transcriptional, DNA, and epigenetic profiles of evolved mutants, as compared to their parentals and among themselves. It would be also of interest to determine how high increases of MICs are related to dosage compensation and increased histone H4 acetylation that were previously demonstrated on the monosomic Ch5 [25,26].

### 3.3. Medical Importance

If caspofungin-adapted mutants with Ch5 aneuploidies indeed arise during caspofungin therapy of *C. albicans* infections, our results suggest that these mutants could, under prolonged treatment with this drug, produce the mutants that are highly tolerant to all ECNs. This might imply that combinational therapy with more than one ECN [27] may be more effective than, for example, caspofungin only.

## 4. Conclusions

We find that initial adaptation of *C. albicans* to caspofungin on agar plates exhibits at least two distinct patterns: simultaneous increase of tolerance to all three clinically relevant ECNs or to caspofungin only. This implies that tolerance to different ECNs can depend either on individual mechanisms or on common mechanism.

We also find that the response of caspofungin-adapted mutants to further challenge by this drug depends on the mutant class. For example, mutants with Ch5 aneuploidy predominantly acquire high tolerance to all three ECNs, upon further exposure to caspofungin. However, mutants with no ploidy change typically acquire only moderate increases in cross-adaptation to other ECNs.

## Figures and Tables

**Table 1:** Caspofungin-adapted parentals and their micafungin and anidulafungin phenotypes *.

Strain	CAS	MFG **	ANI **
No ploidy change			
Parent JRCT1 JMC200-2-5 JMC160-2-5	− + +	− + +	− + +
Ch5 monosomic			
Parent SC5314 SMC60-3-4 SMC60-2-5	− + +	− − − − −	− − − − −
Iso-Ch5R			
Parent JRCT1 JMC120-1-6 JMC120-2-5	− + +	− − − − −	− INC INC

* − or + refer to, respectively, susceptibility or tolerance. − − refers to increased susceptibility. ** See Figure S2A for the growth in presence of micafungin or anidulafungin. INC stands for inconsistent growth.

**Table 2:** Increase of MICs of colonies that evolved from various classes of caspofungin-adapted mutants * as determined with broth microdilution assay. Note that each evolved mutant was validated for adaptation to caspofungin followed by cross-adaptation to other ECNs. Additionally, note a non-random distribution of dramatically increased MICs that are in bold.

D			CAS			MFG			ANI		
CAS-Adapted Parental Mutant *	2X MIC Conc. (µg/mL)	No. of Recove-red Cols.	No. of Evolved Colonies—Increase of MICs	No. of Colonies W. 16- or 32-Fold Increase of MICs	% of Colonies w. 16- or 32-Fold Increase of MICs	No. of Evolved Colonies—Increase of MICs	No. of Colonies W. 16- or 32-Fold Increase of MICs	% of Colonies W. 16- or 32-Fold Increase of MICs	No. of Evolved Colonies—Increase of MICs	No. of Colonies W. 16- or 32-Fold Increase of MICs	% of Colonies W. 16- or 32-Fold Increase of MICs **
JMC200-2-5 No ploidy change	0.500	26	3–4-fold 1–8-fold	0	NA	1–2-fold	0	NA	1–2-fold	0	NA
JMC160-2-5 No ploidy change	0.500	26	4–2-fold 5–4-fold 2–8-fold **1–128-fold**	1	3.8%	0	0	NA	0	0	NA
JMC120-2-5 iso-Ch5	0.125	5	3–8-fold	0	NA	**2–32-fold** **1–128-fold**	3	60%	**2–16-fold** **1–64-fold**	3	60%
JMC120-1-6 iso-Ch5R	0.125	17	2–4-fold 2–8-fold **1–16-fold** **3–32-fold** **2–64-fold**	6	35%	2–4-fold, 1–8-fold **1–16-fold** **5–64-fold**	6	35%	1–4-fold, 2–8-fold **3–16-fold** **2–32-fold** **1-64-fold**	6	35%
SMC60-3-4 Ch5 mono	0.250	12	2–2-fold **3–16-fold** **4–32-fold** **2–64-fold**	9	75%	1–4-fold 1–8-fold **4–16-fold** **1–32-fold** **1–64-fold** **1–128-fold**	7	58.3%	1–8-fold **3–16-fold** **1–64-fold** **3–128-fold** **1–256-fold**	8	66.7%
SMC60-2-5 Ch5 mono	0.250	20	2–2-fold 2–4-fold 1–8-fold **2–16-fold** **3–32-fold**	5	25%	1–8-fold **3–16-fold** **1–32-fold** **2–64-fold** **2–128-fold**	8	40%	2–4-fold 1–8-fold **1–32-fold** **2–128-fold** **3–256-fold**	6	30%

* For the origin of caspofungin-adapted mutants see Table S1. ** Each strain was seeded as 3 technical replicates (~10^8^ colony forming units/plate) on YPD solid medium supplemented with CAS. After the number of colonies with increased MIC was determined for each of 3 plates, the frequencies were calculated for each plate and subsequently averaged. CAS, MFG, ANI—refers to caspofungin, micafungin, anidulafungin, correspondingly. NA—Not Applicable. Additionally, see Figure 3 and Figure S3.

## Data Availability

Not applicable.

## Supplementary Materials

The following supporting information can be downloaded at: https://www.mdpi.com/article/10.3390/microorganisms11010023/s1.
