# Supplementary material for: Candida albicans Strains Adapted to Caspofungin Due to Aneuploidy Become Highly Tolerant under Continued Drug Pressure
Source: Microorganisms. 2022 Dec 21;11(1):23. doi: 10.3390/microorganisms11010023 (PMC9866909; doi:10.3390/microorganisms11010023)
Supplement: Supplementary file 1 [file microorganisms-11-00023-s001.zip › microorganisms-2120777-supplementary.pdf]

Supplemental Materials:

Table S1. *C. albicans* caspofungin-adapted mutants that are used as parentals and their progenitor strains JRCT1 and SC5314.

Strain	Description	Caspofungin MIC (μg/ml)	Source
**JRCT1**	Progenitor strain, diploid	0.031	[13]
**JMC200-2-5**	Same as JRCT1, but caspofungin-adapted	0.250	Same
**JMC160-2-5**	Same as JRCT1, but caspofungin-adapted	0.250	Same
**JMC120-1-6**	Same as JRCT1, but iso-Ch5R, caspofungin-adapted	0.062	Same
**JMC120-2-5**	Same as JRCT1, but iso-Ch5R, caspofungin-adapted	0.062	Same
**SC5314**	Progenitor strain, diploid	0.062	Same
**SMC60-3-4**	Same as SC5314, but Ch5 mono, caspofungin-adapted	0.125	Same
**SMC60-2-5**	Same as SC5314, but Ch5 mono, caspofungin-adapted	0.125	Same

Table S2. List of mutants that evolved from caspofungin-adapted parental strains in the presence of caspofungin. Also listed are caspofungin-adapted parentals and MICs of all strains that were measured as 90% growth inhibition relative to a drug free control (see Figures S1 and S2).

CAS-adapted parental	CAS-evolved mutant	CAS MIC_{90} (μg/ml)/fold change	MFG MIC_{90} (μg/ml)/fold change	ANI MIC_{90} (μg/ml)/fold change
**JMC200-2-5**<br>**No ploidy change**<br>**CAS MIC** 0.03 μg/ml<br>**MFG MIC** 0.03 μg/ml<br>**ANI MIC** 0.06 μg/ml	B1	0.12/4-fold	0.06/2-fold	0.12/2-fold
	B2	0.12/4-fold	0.03/No change	0.06/No change
	B3	0.12/4-fold	0.03/No change	0.03/No change
	B5	0.25/8-fold	NA	NA
**JMC160-2-5**<br>**No ploidy change**<br>**CAS MIC** 0.03 μg/ml	B2-1	0.25/8-fold	NA	NA
	B2-2	0.12/4-fold	NA	NA
	B2-4	0.12/4-fold	NA	NA
	B2-7	0.06/2-fold	NA	NA
	B2-8	0.06/2-fold	NA	NA
	B2-10	0.06/2-fold	NA	NA
	B2-11	0.12/4-fold	NA	NA
	B2-14	>4.0/128-fold*	NA	NA
	B2-16	0.06/2-fold	NA	NA
	B2-20	0.25/8-fold	NA	NA
	B2-22	0.12/4-fold	NA	NA
	B2-23	0.12/4-fold	NA	NA
**JMC120-2-5**<br>**iso-Ch5R**<br>**CAS MIC** 0.06 μg/ml<br>**MFG MIC** 0.03 μg/ml	C1	0.5/8-fold	1.0/32-fold	0.500/16-fold
	C2	0.5/8-fold	1.0/32-fold	0.500/16-fold
	C5	0.5/8-fold	>4.0/128-fold	2.0/64-fold

**ANI MIC** 0.03 µg/ml				
**JMC120-1-6**	C2-1	2.00/32-fold	4.0/64-fold	2.0/32-fold
**iso-Ch5R**	C2-6	0.5/8-fold	4.0/64-fold	2.0/32-fold
	C2-7	1.0/16-fold	NA	NA
**CAS MIC** 0.06 µg/ml	C2-8	0.25/4-fold	0.25/4-fold	0.5/8-fold
**MFG MIC** 0.06 µg/ml	C2-9	2.0/32-fold	0.5/8-fold	0.5/8-fold
**ANI MIC** 0.06 µg/ml	C2-10	>4.0/64-fold	1.0/16-fold	>4.0/64-fold
	C2-11	0.5/8-fold	4.0/64-fold	1.0/16-fold
	C2-12	0.25/4-fold	0.25/4-fold	0.25/4-fold
	C2-13	2.0/32-fold	>4.0/64-fold	1.0/16-fold
	C2-14	>4.0/64-fold	>4.0/64-fold	1.0/16-fold
**SMC60-3-4**	E2	1.0/16-fold	0.5/16-fold	0.25/16-fold
**Ch5 monosomy**	E3	1.0/16-fold	0.12/4-fold	4.0/256-fold
	E4	1.0/16-fold	0.5/16-fold	0.25/16-fold
**CAS MIC** 0.06 µg/ml	E5	2.0/32-fold	4.0/128-fold	2.0/128-fold
**MFG MIC** 0.03 µg/ml	E6	2.0/32-fold	0.5/16-fold	0.25/16-fold
**ANI MIC** 0.01 µg/ml	E7	2.0/32-fold	0.5/16-fold	1.0/64-fold
	E8	0.12/2-fold	NA	NA
	E9	4.0/64-fold	2.0/64-fold	2.0/128-fold
	E10	2.0/32-fold	1.0/32-fold	2.0/128-fold
	E11	4.0/64-fold	0.25/8-fold	0.12/8-fold
	E12	0.12/2-fold	NA	NA
**SMC60-2-5**	E2-1	2.0/16-fold	2.0/64-fold	4.0/256-fold
**Ch5 monosomy**	E2-2	0.12/ No change	NA	NA
	E2-3	0.25/2-fold	NA	NA
**CAS MIC** 0.12 µg/ml	E2-4	>4.0/32-fold	0.25/8-fold	4.0/256-fold
	E2-5	0.5/4-fold	2.0/64-fold	0.06/4-fold

**MFG MIC** 0.03 μg/ml<br>**ANI MIC** 0.01 μg/ml	E2-6	>4.0/32-fold	4.0/128-fold	2.0/128-fold
	E2-7	0.5/4-fold	0.5/16-fold	0.5/32-fold
	E2-8	1.0/8-fold	0.5/16-fold	4.0/256-fold
	E2-9	0.12/ No change	1.0/32-fold	0.06/4-fold
	E2-10	2.0/16-fold	0.5/16-fold	2.0/128-fold
	E2-11	0.25/2-fold	NA	NA
	E2-12	0.06/ No change	NA	NA
	E2-13	0.06/ No change	NA	NA
	E2-14	0.12/ No change	NA	NA
	E2-15	0.12/ No change	NA	NA
	E2-16	0.06/ No change	NA	NA
	E2-17	>4.0/32-fold	4.0/128-fold	0.12/8-fold
	E2-18	>0.06/ No change	NA	NA
	E2-19	0.12/ No change	NA	NA
	E2-20	0.12/ No change	NA	NA

*Note that this evolved mutant showed very clear inhibition of growth at concentration of 0.25 μg/ml, which is 8-fold increase (see Fig S2A). However, it showed ~10% of growth at every higher concentration of caspofungin. NA stands for Not Available.

Primer name and purpose	Sequence
**Amplification of HS1 and HS2 regions of *FKS1***	
HS1-A, F	CGGTGCTCAACATTTGAGTCGTCGTAT
HS1-A, R	TTGATTTCCATTTCCGTGGTAGCTAAA
HS2-A, F	TGCTGGTATGAATGCCATGATGAGAGG
HS2-A, R	GGTGCTTGCCAATGAGAAACTGTACCA
**Sequencing of HS1 and HS2 regions of *FKS1***	
HS1-S, F	CGGCATATGCTGTGTCGATTGT
HS1-S, R	TGAACGACCAATGGAGAAGA
HS2-S, F	TTGGTGCTGGTATGGGAGAACA
HS2-S, R	GCACCACCAACGGTCAAATCA

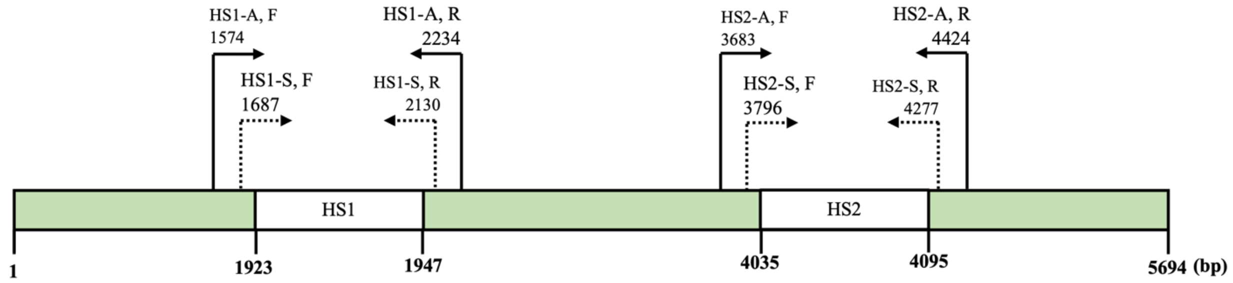

Figure S1. Primers and sequences. Shown below is a cartoon of *C. albicans FKS1* gene showing the hot spot regions HS1 and HS2. Solid arrows represent the amplification primers and dashed arrows represent the sequencing primers.

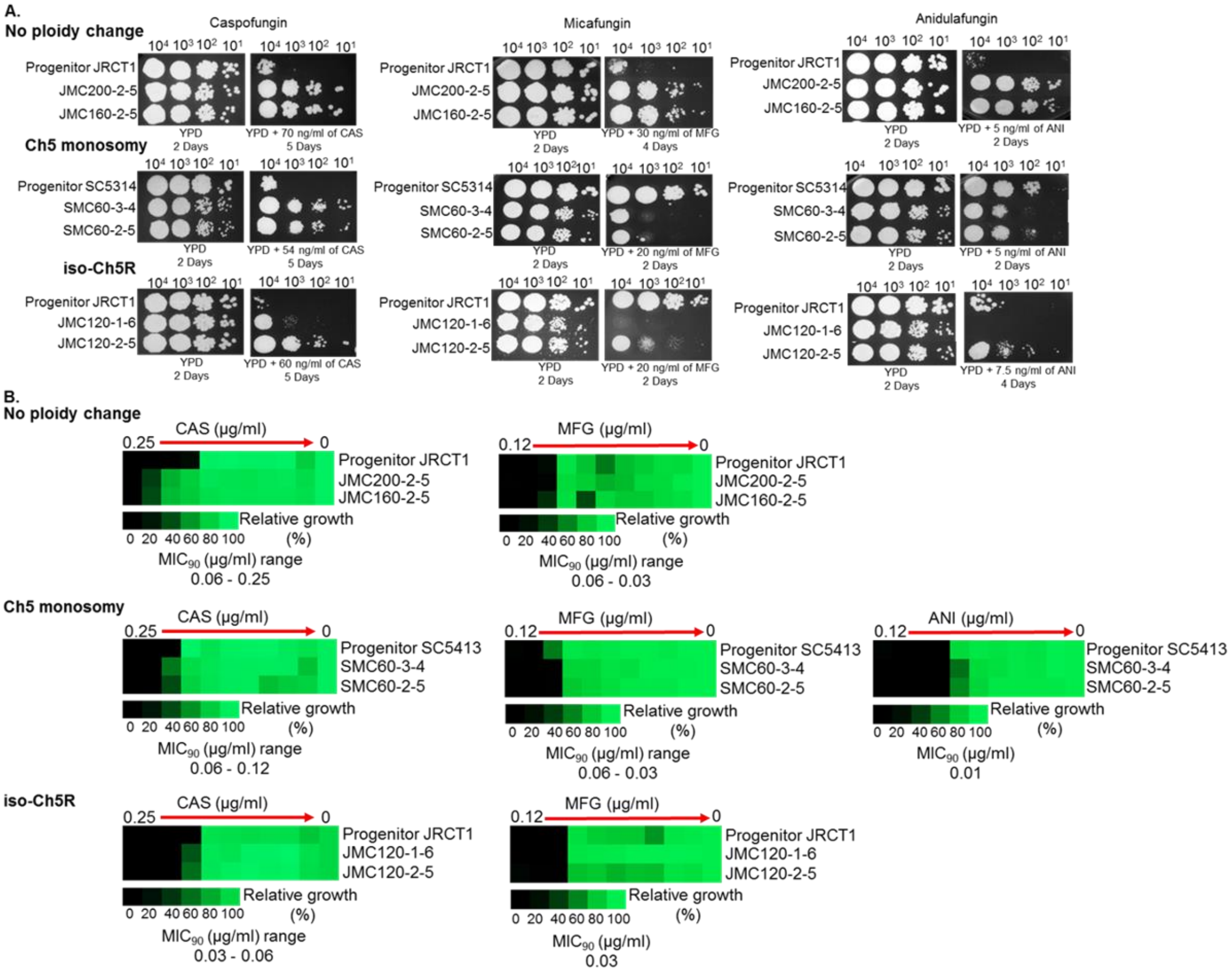

Figure S2. Different classes of caspofungin-adapted parental mutants exhibit different echinocandin (ECN) susceptibilities. CAS, MIC and ANI refer to caspofungin, micafungin and anidulafungin, respectively. **A**. The spot assay shows difference of growth on YPD medium supplemented with ECN drugs, as indicated. The plates were incubated at 37 ºC. The names of progenitor strains JRCT1 or SC5314, caspofungin-adapted parental mutants and Ch5 condition (no ploidy change, Ch5 monosomy and iso-Ch5R) are indicated on left. Dilution factor, ECNs concentration and days of plate incubation are indicated on top and bottom of spot assays images, respectively. **B**. The heat maps show the growth of the strains in (A) by broth microdilution method in the presence of ECN drugs, as indicated. The assay was conducted

according to CLSI method in YPD medium with maximum concentration of CAS, MIC and ANI of 0.250 μg/ml, 0.125 μg/ml & 0.125 μg/ml respectively, with 2-fold serial dilution. A total of 10^3 cells were inoculated into each well of 96-well plates in triplicates or quadruplicates (technical replicates) and plates were incubated at 35 °C for 48h. Control wells without drug or without cells were included. The no-cell control was used to subtract the background. The no-drug control was used for normalization. MICs were determined as 90% growth inhibition relative to a drug free control. The color bar for percent growth is presented below each panel. Note that spot assay in (A) resolved unclear MICs for MFG and ANI by broth microdilution in (B).

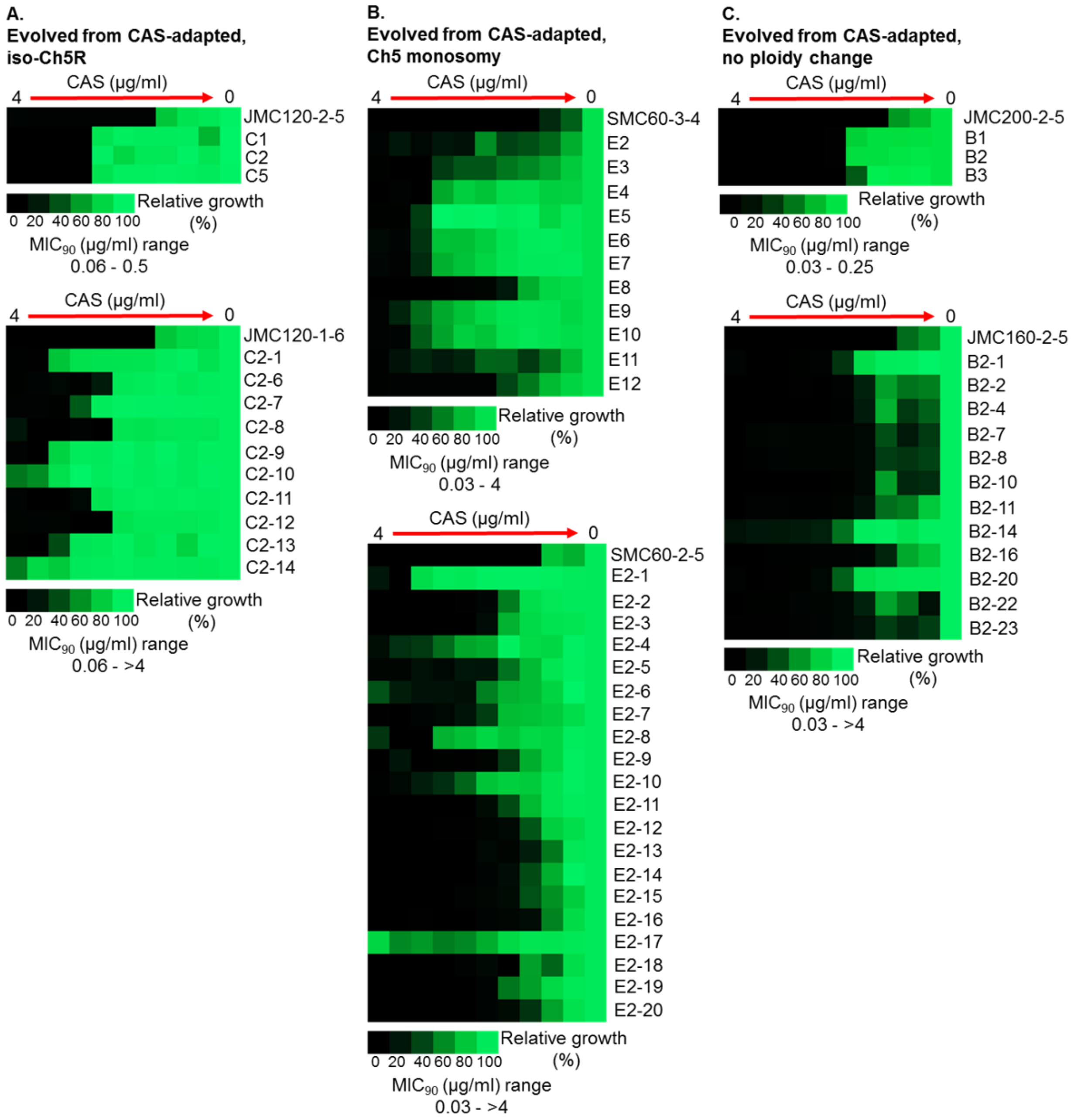

Figure S3a. Caspofungin-evolved mutants exhibit increased caspofungin MICs, as presented with the heat maps. Shown are derivatives of parentals with iso-Ch5R, Ch5 monosomy or no ploidy change as indicated. Names of strains are indicated on the left. Broth microdilution assay was performed according to CLSI method in YPD medium including maximum CAS concentration of 4 μg/ml. For more details, see Figure S2B legend. Color bar for % growth is

presented for each panel underneath. MICs were measured as 90% growth inhibition relative to a drug free control.

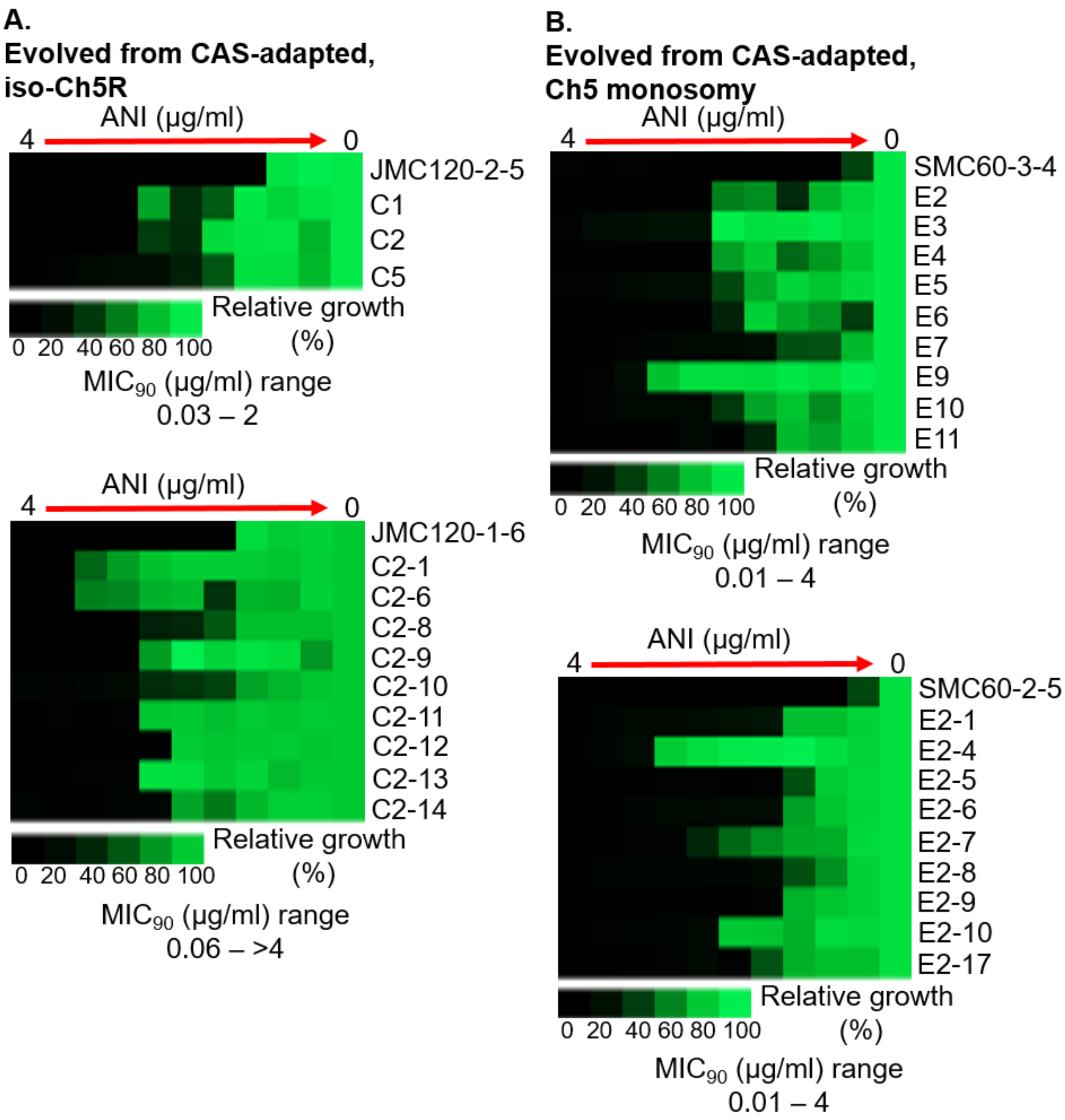

Figure S3b. Caspofungin-evolved mutants exhibit increased anidulafungin MICs, as shown with the heat maps. Shown are derivatives of parentals with iso-Ch5R and Ch5 monosomy. For more details, see legends of Figures S2B and S3a. Note that derivatives of no ploidy change strains showed no change of ANI MICs.

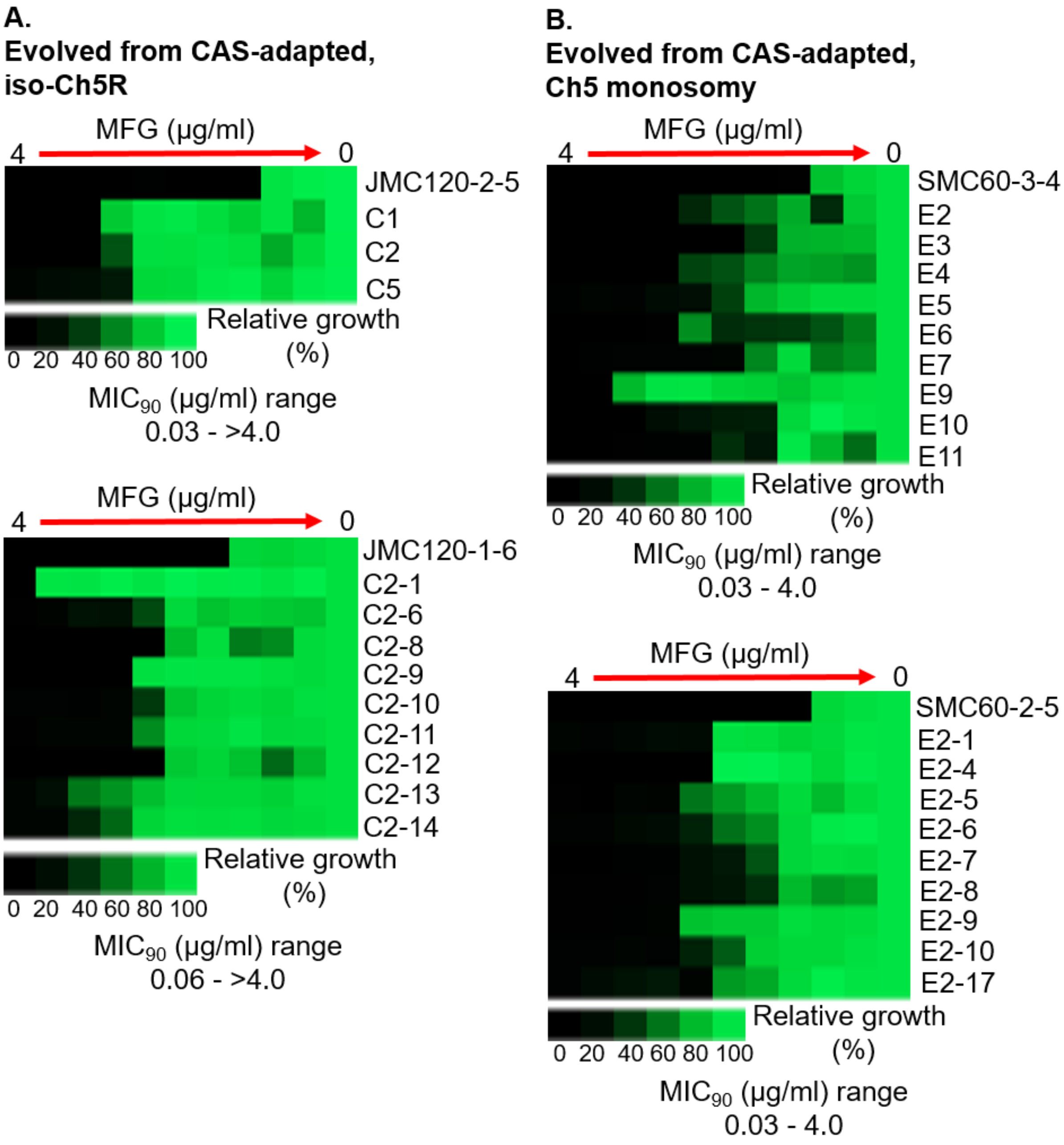

Figure S3c. Caspofungin-evolved mutants exhibit increased micafungin MICs, as shown with the heat maps. Shown are derivatives of parentals with iso-Ch5R and Ch5 monosomy. For more details, see legends of Figures S2B and S3a.
